# An intervention in contraceptive counseling increased the knowledge about fertility and awareness of preconception health—a randomized controlled trial

**DOI:** 10.1080/03009734.2019.1653407

**Published:** 2019-09-09

**Authors:** Yvonne Skogsdal, Helena Fadl, Yang Cao, Jan Karlsson, Tanja Tydén

**Affiliations:** a Maternal Health Care Unit, Faculty of Medicine and Health, Örebro University, Örebro, Sweden;; b Department of Obstetrics and Gynecology, Faculty of Medicine and Health, Örebro University, Örebro, Sweden;; c Clinical Epidemiology and Biostatistics, School of Medical Sciences, Örebro University, Örebro, Sweden;; d University Health Care Research Center, Faculty of Medicine and Health, Örebro University, Örebro, Sweden;; e Department of Women’s and Children’s Health, Akademiska Sjukhuset, Uppsala, Sweden

**Keywords:** Contraceptive counseling, fertility, lifestyle factors, preconception care, preconception health, pregnancy, reproductive life plan

## Abstract

**Background:** Reproductive life plan counseling (RLPC) is a tool to encourage women and men to reflect upon their reproduction, to avoid unintended pregnancies and negative health behavior that can threaten reproduction. The aim was to evaluate the effect of RLPC among women attending contraceptive counseling. Outcomes were knowledge about fertility and awareness of preconception health, use of contraception, and women’s experience of RLPC.

**Material and methods:** Swedish-speaking women, aged 20–40 years, were randomized to intervention group (IG) or control group (CG). Participants (*n* = 1,946) answered a questionnaire before and two months after (*n* = 1,198, 62%) the consultation. All women received standard contraceptive counseling, and the IG also received the RLPC, i.e. questions on reproductive intentions, information about fertility, and preconception health.

**Results:** Women in the IG increased their knowledge about fertility: age and fertility, chances of getting pregnant, fecundity of an ovum, and chances of having a child with help of IVF. They also increased their awareness of factors affecting preconception health, such as to stop using tobacco, to refrain from alcohol, to be of normal weight, and to start with folic acid before a pregnancy. The most commonly used contraceptive method was combined oral contraceptives, followed by long-acting reversible contraception. Three out of four women (76%) in the IG stated that the RLPC should be part of the routine in contraceptive counseling.

**Conclusions:** Knowledge about fertility and awareness of preconception health increased after the intervention. The RLPC can be recommended as a tool in contraceptive counseling.

## Introduction

Preconception health and care are of growing interest as they can affect fertility, pregnancy outcomes, and the health of the offspring both in the short and in the long term (1–4). Lifestyle factors such as smoking, obesity, and alcohol consumption have a negative impact on fertility and pregnancy outcomes. When planning a pregnancy, it would be optimal to change an unhealthy lifestyle to a healthier one in order to improve one’s outcomes. There are preconception guidelines for women with chronic diseases, but only fragmentary guidelines exist for healthy women (5). An increased intake of folate through food or vitamin supplementation is recommended in many countries for all women who might become pregnant, in order to prevent neural tube defects (6). However, in a Swedish study, only 4 out of 10 women took folic acid prior to pregnancy, despite the fact that the pregnancy was very planned (7).

For promotion of preconception health and care, Moos et al. developed a tool called Reproductive Life Plan (RLP). It is intended for both women and men to reflect upon their reproductive intentions, to find strategies for successful family planning, and to avoid unwanted pregnancies and adverse health outcomes that may adversely affect reproduction (8). The RLP focuses on the individual’s goal of having children or not, as well as a plan for how to achieve this goal. Jack et al. recommend that women of fertile age should be screened for their intentions to become or not become pregnant, both from a short- and a long-term perspective, and also screened for their risk of an unwanted pregnancy (9). An RLP can help women to choose a suitable contraceptive method according to their reproductive goals. However, a US study did not find support for the increased use of effective contraceptives following Reproductive Life Plan Counseling (RLPC) among women (10). A Swedish study on female university students found that after RLPC the knowledge of preconception health increased and the women planned to have their last child earlier in life (11). The RLPC has also been used among men with positive results (12). Midwives who used the RLPC considered it as a health-promoting concept, but they emphasized the importance of tactfulness and were aware that social norms influenced decisions regarding childbearing (13). Since screening tools on pregnancy intentions have gained attention, it has been suggested that the use of these tools in clinical settings needs to be further examined in a general population (14–16).

The specific aim of this study, which is part of a planned longitudinal study, was to evaluate the effect of using the RLPC among a representative sample of Swedish-speaking women visiting registered nurse-midwives (RNM) for contraceptive counseling. The main outcomes were knowledge of fertility and awareness of preconception health. Secondary outcomes were use of contraception and women’s experiences of the RLP.

## Material and methods

**Figure 1.**
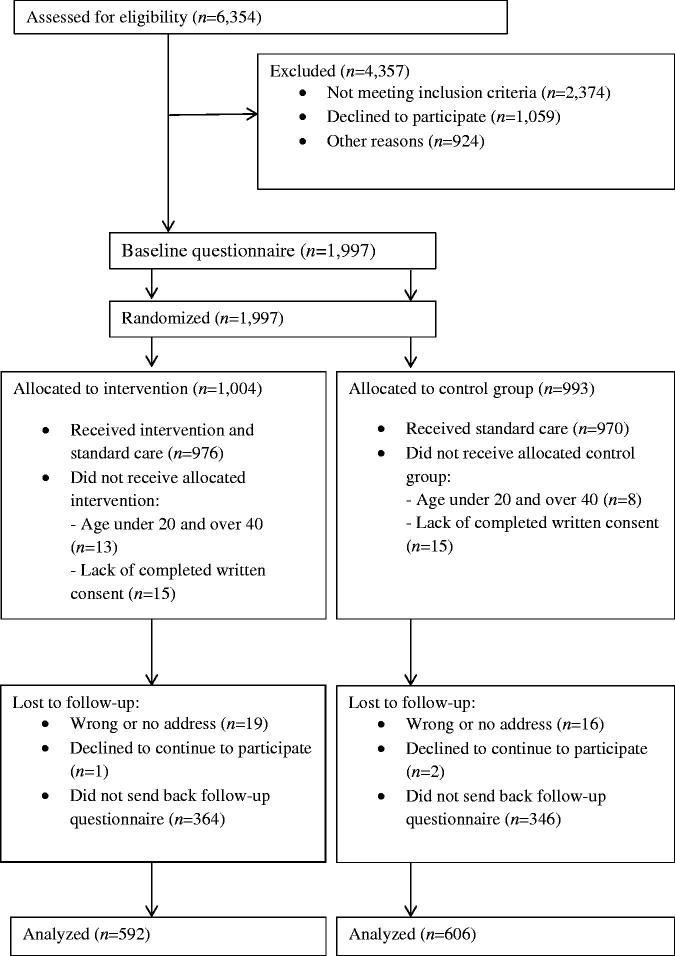
Flow diagram.

A randomized controlled trial was conducted between February 2015 and March 2016. All women (*n* = 6,354) attending contraceptive counseling during the study period were assessed for eligibility. Inclusion criteria were: aged between 20 and 40 years and able to read and understand Swedish. A flow diagram of the study is presented in Figure 1.

### Sample size

In addition to the evaluation of primary and secondary outcomes in the present study, the plan is to follow the participants with their future pregnancies. Accordingly, the sample size calculation is based on both immediate outcomes, as well as on the assumption that the intervention might have a possible effect on future preconception health.

For the present study, assuming that knowledge about folic acid would increase from 20% to 40% after the intervention (11), with a power of 0.8 and a significance level of *P* < 0.05, 82 women would be needed in each group.

For the planned future longitudinal study, assuming that the intervention would affect women to make lifestyle changes before a future pregnancy, the sample size was calculated accordingly: a) to reduce hazardous use of alcohol by 50% (from 6% to 3%); b) to reduce smoking 3 months before the pregnancy by 50% (from 13.5% to 6.75%); and c) to reduce the proportion of women with BMI ≥25 in early pregnancy from 40% to 32%. With a power of 0.8 and a significance level of *P* < 0.05, a) 749, b) 312, and c) 564 would be needed in each group. It was not possible to accurately estimate how many of the women who participated in the study would become pregnant in the future. However, assuming that approximately 50% would become pregnant, it was decided that 1,000 individuals would be included in each group.

### Setting

In Sweden, midwives are licensed to insert contraceptive devices and prescribe hormonal contraceptives to healthy women. A total of 86 midwives at 28 outpatient clinics in central Sweden participated in the data collection.

Before the commencement of the study, the midwives were informed about the study rationale and the intervention to ensure equivalent implementation. They were instructed how to counsel the intervention and control group as well.

As described by Skogsdal et al. (17), women were informed about the study when they called the clinic to make their appointment. Later, when they attended the clinic, they received written and oral information. They were also informed that participation was voluntary and that they could interrupt their participation at any time without explanation. Those who agreed to participate signed an informed consent and were randomly assigned to a control group (CG) or an intervention group (IG). They were informed that the signed consent with their personal identification number should be kept separate from the questionnaire. All data were coded and stored in secure servers in order to minimize risk of unauthorized access.

### Randomization procedure

Randomization was done within each maternal healthcare unit in clusters. Sealed envelopes were prepared with notes for the IG or CG, together with a baseline questionnaire and a prepaid envelope. The midwife opened the envelope containing instructions for either the CG or the IG. Before the counseling, participants completed the baseline questionnaire in the waiting room. The questionnaire was then put into a sealed envelope and handed over to the midwife. Part of the results from the baseline questionnaire has been presented previously (17).

### Intervention

Both the IG and the CG received routine contraceptive counseling according to the Swedish Medical Product Agency (18), as described in Supplement 1 (available online). In addition to the routine counseling, women in the IG also received the RLPC, which was conducted in the similar way as in a study on female university students (11). The midwives had access to a template with discussion points to choose from, depending on the woman’s answer to the first question, ‘Do you wish to have children/more children in the future?’ For those who did not want children/more children, the discussion focused on the importance of choosing effective contraceptives and general information about preconception health. For women who wished to have children, information was focused on preconception health, including folic acid supplementation and use of contraception until a pregnancy was planned. For women who wanted to postpone childbearing for years, the focus was on contraception for a long time, preconception health, and information about fertility and age. For those who were unsure about their reproductive intention, the discussion was about contraception and a healthy lifestyle. The midwife gave the women standardized information based on a checklist (11). They also gave the women in the IG a specially designed 18-page booklet to read. The booklet contained information/facts about fertility and preconception health.

### Questionnaires

The outcome was evaluated using two questionnaires: at baseline (Q1) and at follow-up (Q2) (Questionnaires 1 and 2, available online). The questions were developed on the basis of the previous study of university students (19). Some questions were modified, and some new questions were added. Before the present study, the questionnaire was piloted on 28 women attending contraceptive counseling, whereupon a few questions were modified.

Q1 consisted of 41 multiple-choice questions on background and demographics (Table 1), knowledge about fertility (Table 3), preconception health (Table 4), the use of contraception at latest intercourse (Table 6), including the question ‘How satisfied are you with your current contraceptive?’ in a five-grade response scale, and reproductive intention: ‘Do you want children/more children in the future?’ and ‘How many children do you want?’ We also asked about when they wanted to have their first and last child, as well as the probability of having an unplanned pregnancy in the future.

**Table 1.** Background and demographics among those who answered the baseline questionnaire and comparison between those who answered and did not answer the follow-up questionnaire.

	Answer baseline *n* = 1946	Answer baseline and follow-up *n* = 1204	Answer baseline and not follow-up *n* = 742	*P*
Age, median year (interquartile range)	25 (22, 31)	26 (22, 31)	24 (3.4)	0.003
BMI, *n* (%)				
Underweight (<18.5)	14 (0.7)	26 (2.2)	25 (2.3)	0.263
Normal weight (18.5–24.99)	1198 (64.3)	763 (65.5)	435 (61.1)	
Overweight (25–29.99)	430 (22.0)	259 (22.3)	171 (24.0)	
Obese class I (30–34.99)	138 (7.1)	80 (6.9)	58 (8.1)	
Obese class II (>35)	47 (2.4)	36 (3.1)	24 (3.4)	
Education, *n* (%)				
Non-completed education (<9 years)	14 (0.7)	4 (0.3)	10 (1.3)	<0.001
Elementary school (9 years)	82 (4.2)	33 (2.8)	49 (6.6)	
High school (12 years)	1145 (58.9)	678 (56.6)	467 (62.7)	
Professional education (non-academic)	159 (21.3)	99 (8.3)	60 (8.1)	
College/university	543 (27.8)	384 (32.1)	159 (8.1)	
Main occupation, *n* (%)				
Working	1105 (56.9)	644 (53.8)	461 (61.8)	<0.001
Student	535 (27.5)	361 (30.2)	174 (23.3)	
Parental leave	171 (8.8)	123 (10.3)	48 (6.4)	
Unemployed	66 (3.4)	32 (2.7)	34 (4.6)	
Sick leave	52 (2.7)	28 (2.3)	24 (3.2)	
Other	14 (0.7)	9 (0.8)	5 (0.7)	
Country of birth, *n* (%)				
Sweden	1798 (92.9)	1129 (94.7)	669 (90.0)	0.002
Other Nordic country	14 (0.7)	6 (0.5)	8 (1.1)	
Other European country	50 (2.6)	25 (2.1)	25 (3.4)	
Outside Europe	74 (3.8)	33 (2.8)	41 (5.5)	
Sexual orientation, *n* (%)				
Heterosexual	1823 (94.4)	1135 (95.1)	688 (93.2)	0.109
Bisexual	80 (4.1)	47 (3.9)	33 (4.5)	
Homosexual	1 (0.1)	1 (0.1)	0 (0.0)	
Do not know/unsure	21 (1.1)	9 (0.8)	12 (1.6)	
Other	7 (0.4)	2 (0.2)	5 (0.7))	
Stable relationship, *n* (%)	1476 (76.3)	927 (77.7)	549 (74.0)	0.062
Reproduction, *n* (%)				
Had tried to get pregnant	652 (33.7)	412 (34.5)	240 (32.4)	0.348
Had been pregnant	881 (45.4)	512 (42.8)	369 (49.6)	0.003
Had given birth	656 (75.5)	413 (81.5)	243 (67.1)	<0.001
Experience of abortion	280 (32.3)	135 (26.7)	145 (40.1)	<0.001
Experience of miscarriage	112 (12.9)	78 (15.4)	34 (9.4)	0.009
Smoking, *n* (%)				
Smoking daily	199 (10.2)	102 (8.6)	97 (13.1)	<0.001
Smoking, but not daily	221 (11.5)	108 (9.1)	113 (15.3)	
Former smoker	429 (22.0)	276 (23.2)	153 (20.7)	
Never smoked	1077 (55.9)	702 (59.1)	375 (50.8)	
Swedish snuff, *n* (%)				
Snuff daily	126 (6.4)	65 (5.5)	61 (8.3)	0.013
Use snuff, but not daily	81 (4.2)	47 (4.0)	34 (4.6)	
Former user of snuff	157 (8.1)	93 (7.8)	64 (8.7)	
Never used snuff	1563 (81.1)	984 (82.8)	579 (78.5)	
Drinking alcohol, 4 standard glasses or more at the same time, *n* (%)^a^				
Daily	1 (0.1)	1 (0.1)	0 (0)	0.073
Once/week	94 (4.8)	54 (4.5)	40 (5.5)	
Once/month	495 (25.8)	290 (24.3)	205 (28.0)	
Less than once/month	972 (49.7)	618 (51.9)	354 (48.4)	
Never	360 (18.7)	228 (19.1)	132 (18.1)	

a One standard glass is: Beer (<3.5%) 50 cL; beer (>3.5%) 33 cL; wine (8%–15%) 12–15 cL; wine (15%–22%) 8 cL; or liquor 4 cL.

Q2 was sent by post, two months after the counseling, with two reminders. Most questions in Q2 were repeated from Q1 (Table 2). We also asked them: ‘In the last 2 months, have you at any time talked to someone close to you, for example, your partner or a friend, about fertility (ability to become pregnant)?’ and ‘What did you talk about?’ The IG also received additional questions about the experiences of the RLPC: ‘Did you read the brochure you received from the midwife?’ ‘What did you think when the midwife asked you about your reproductive life plan?’ and ‘Do you think that the midwife or other categories of healthcare professionals, e.g. doctors, should have as a routine to discuss the Reproductive Life Plan with their patients?’

**Table 2.** Demographic background of study population answering Q2.

	Q2 Intervention	Q2 Control	*P*
Age, median year (interquartile range)	25 (22, 31)	26 (23, 31)	0.518
BMI, *n* (%)			
Underweight (<18.5)	12 (2.1)	14 (2.4)	0.037
Normal weight (18.5–24.99)	359 (62.7)	404 (68.4)	
Overweight (25–29.99)	140 (24.4)	119 (20.1)	
Obese class I (30–34.99)	44 (7.7)	36 (6.1)	
Obese class II (>35)	18 (3.1)	18 (3.0)	
Education, *n* (%)			
Non-completed education (<9 years)	3 (0.5)	1 (0.2)	0.088
Elementary school (9 years)	16 (2.7)	17 (2.8)	
High school (12 years)	349 (59.0)	329 (54.3)	
Professional education (non-academic)	47 (7.9)	52 (8.6)	
College/university	177 (29.9)	207 (34.2)	
Main occupation, *n* (%)			
Working	315 (53.2)	329 (54.4)	0.882
Student	184 (31.1)	177 (29.3)	
Parental leave	60 (10.1)	63 (10.4)	
Unemployed	13 (2.2)	19 (3.1)	
Sick leave	15 (2.5)	13 (2.1)	
Other	5 (0.8)	4 (0.7)	
Country of birth, *n* (%)			
Sweden	557 (94.7)	572 (94.5)	0.291
Other Nordic country	1 (0.2)	5 (0.8)	
Other European country	11 (1.9)	14 (2.3)	
Outside Europe	19 (3.2)	14 (2.8)	
Sexual orientation, *n* (%)			
Heterosexual	656 (95.6)	570 (94.5)	0.719
Bisexual	22 (3.7)	25 (4.1)	
Homosexual	0 (0.0)	1 (0.2)	
Do not know/unsure	3 (0.5)	6 (1.0)	
Other	1 (0.2)	1 (0.2)	
Stable relationship, *n* (%)	453 (76.8)	474 (78.6)	0.448
Reproduction, *n* (%)			
Had tried to get pregnant	209 (35.3)	203 (33.7)	0.551
Had been pregnant	255 (43.1)	257 (42.4)	0.796
Had given birth	208 (81.9)	205 (81.0)	0.803
Experience of abortion	65 (25.7)	70 (27.7)	0.615
Experience of miscarriage	43 (17)	35 (13.8)	0.325
Smoking, *n* (%)			
Smoking daily	54 (9.2)	48 (8.0)	0.034
Smoking, but not daily	65 (11.0)	43 (7.2)	
Former smoker	138 (23.4)	138 (23.0)	
Never smoked	332 (56.4)	370 (61.8)	
Swedish snuff, *n* (%)			
Snuff daily	30 (5.1)	35 (5.8)	0.881
Use snuff, but not daily	20 (3.4)	27 (4.5)	
Former user of snuff	53 (9.0)	40 (6.6)	
Never used snuff	115 (82.4)	113 (83.1)	
Drinking alcohol, 4 standard glasses or more at the same time, *n* (%)^a^			
Daily	1 (0.2)	0 (0)	0.416
Once/week	28 (4.8)	26 (4.3)	
Once/month	133 (22.7)	157 (26.0)	
Less than once/month	310 (52.8)	308 (51.0)	
Never	115 (19.6)	113 (18.7)	

a One standard glass is: Beer (<3.5%) 50 cL; beer (>3.5%) 33 cL; wine (8%–15%) 12–15 cL; wine (15%–22%) 8 cL; or liquor 4 cL.

The CG received additional questions about if they had talked with the midwife about ‘Having children in the future’, ‘Importance of age for fertility’, ‘Probability of becoming pregnant after an unprotected sexual intercourse’, ‘Factors that can affect the fertility’, ‘Factors that can increase the probability of having a healthy pregnancy’, ‘Chances of having a baby via IVF’, and the ‘Lifespan of ovum and sperm’.

### Statistical analysis

Participants’ demographic characteristics and responses are presented in percentages, proportions, or median with a corresponding interquartile range wherever suitable.

Chi-square or Fisher’s exact tests were used for categorical variables. Mann–Whitney *U* test was used for ordinal variables and asymmetrically distributed continuous variables. The difference in the response rate between the groups was tested using Mantel–Haenszel chi-square test, adjusting for the same question in the first questionnaire. The association between the intervention and the participants’ responses was also presented using relative risk (RR), with a corresponding 95% confidence interval (CI).

SPSS Statistics version 22 (IBM Corp., Armonk, NY, USA) was used in all statistical analyses. In all of the analyses, two-sided *P* values <0.05 were considered statistically significant.

### Ethical approval

The Regional Ethical Review Board in Uppsala, Sweden approved the study (Dnr 2012/101).

The study was registered in ISRCTN 32759.

## Results

In total, after exclusions, 1,946 women answered Q1 and 1,198 Q2 (CG, *n* = 606; and IG, *n* = 592), which is a response rate of 62%. The internal response rate was high for most questions in both questionnaires (95.5%–100%). Non-responders to Q2 had lower education, and a greater proportion were smokers, users of snuff, workers, and born abroad. They also had different experiences of pregnancies (Table 1).

There were more women with overweight and more smokers in the IG. There were no other differences between the IG and the CG regarding the background characteristics (Table 2).

### Fertility knowledge

At baseline, knowledge about fertility was low, and 64% (756/1,174) of the participants thought that the probability of a 25-year-old woman becoming pregnant if she had unprotected intercourse with a man of the same age was 60% or more (the correct answer is 30%–35%). A total of 16.7% (195/1,165) answered correctly that the fecundity of an ovum is around 1 day, and 27% (314/1,165) thought it was 5 days or more. The responses to when a woman’s fertility markedly begins to decline varied between 1 and 65 years, with a mean of 35.5 years (the correct answer was around 35 years with individual fluctuations). The chance of giving birth after one attempt of *in vitro* fertilization is 25%–30%, and 22.3% (256/1,144) answered correctly, while 51.9% (594/1,144) thought the chance was 50% or more.

After the intervention, women in the IG increased their knowledge on all questions about fertility (*P* < 0.05; Table 3).

**Table 3.** Percentage of women in the intervention group and the control group who responded correctly or almost correctly to questions about fertility.

	Before		After			
	Intervention	Control	Intervention	Control		
	*n* (%)	*n* (%)	*n* (%)	*n* (%)	Adjusted RR	95% CI
How likely is it for a 25-year-old woman to become pregnant if she has unprotected intercourse with a man at the same age at the time of ovulation? (Correct or almost correct 25%–40%)	69 (11.9)	66 (11.1)	273 (46.5)	121 (20.1)	2.297	1.915–2.755
For how long is it possible for an ovum to be fertilized? (Correct or almost correct 1–2 days)	182 (33.7)	199 (36.0)	306 (54.3)	244 (42.4)	1.245	1.098–1.412
At what age does a woman’s ability to become pregnant begin to decline? (Correct or almost correct 33–37 years)	203 (34.6)	181 (30.2)	300 (50.8)	199 (33.2)	1.541	1.341–1.771
What are the chances of giving birth to a child after *in vitro* fertilization per attempt? (Correct or almost correct 20%–35%)	177 (31.5)	182 (31.3)	306 (52.9)	228 (39.0)	1.354	1.190–1.540

RR was adjusted for the same question before the intervention.

### Awareness of preconception factors

At baseline, to quit smoking before a pregnancy was stated as being the most important. Importance of taking folic acid was unknown for 28%, and nearly 30% thought it was neither important nor unimportant or rather/very unimportant. More parous (69%) than nulliparous (43%) stated that taking folic acid was very or fairly important at baseline (Table 4).

**Table 4.** Women’s awareness of preconception health before and after the intervention.

	Before		After		
	Intervention	Control	Intervention	Control	
	*n* (%)	*n* (%)	*n* (%)	*n* (%)	*P*
How important is it to stop smoking before pregnancy?					
Very and fairly important	557 (94.2)	575 (95.4)	573 (97.6)	574 (94.9)	0.019
Very and fairly unimportant, neither important/unimportant	26 (4.4)	24 (4.0)	14 (2.4)	27 (4.5)	
Do not know	8 (1.4)	4 (0.7)	0 (0)	4 (0.7)	
How important is it to stop using snuff before pregnancy?					
Very and fairly important	525 (88.8)	552 (91.5)	548 (93.4)	530 (88.0)	0.004
Very and fairly unimportant, neither important/unimportant	50 (8.5)	46 (7.6)	33 (5.6)	55 (9.1)	
Do not know	16 (2.7)	5 (0.8)	6 (1.0)	17 (2.8)	
How important is it to start taking folic acid before pregnancy?					
Very and fairly important	261 (44.2)	255 (42.4)	458 (78)	330 (54.5)	<0.001
Very and fairly unimportant, neither important/unimportant	168 (28.4)	180 (29.9)	73 (12.4)	165 (27.3)	
Do not know	162 (27.4)	167 (27.7)	56 (9.5)	110 (18.2)	
How important is it to be of normal weight before pregnancy?					
Very and fairly important	468 (79.2)	460 (76.2)	533 (90.8)	510 (84.7)	0.003
Very and fairly unimportant, neither important/unimportant	10 (17.4)	114 (18.9)	50.(8.5)	80 (13.3)	
Do not know	20 (3.4)	30 (5.0)	4 (0.7)	12 (2.0)	
How important is it to refrain from alcohol before pregnancy?					
Very and fairly important	468 (79.2)	460 (76.2)	507 (86.6)	441 (73.0)	<0.001
Very and fairly unimportant, neither important/unimportant	103 (17.4	114 (18.9)	69 (11.8)	147 (24.3)	
Do not know	20 (3.4)	20 (3.4)	11 (1.9)	16 (2.6)	
How important is it to start taking vitamin C?					
Very and fairly important	133 (22.5)	127 (21.1)	261 (44.8)	164 (27.1)	<0.001
Very and fairly unimportant, neither important/unimportant	311 (54.6)	319 (53.1)	258 (44.3)	328 (54.2)	
Do not know	135 (22.9)	155 (25.8)	64 (11.0)	113 (18.7)	

The answer options in each question was ‘very important’, ‘fairly important’, ‘neither important/unimportant’, ‘fairly unimportant’, ‘very unimportant’, and ‘do not know’. In this table, the response options are congregated.

*P* value was adjusted for the same question before the intervention.

After the intervention a larger proportion of women in the IG thought that it was more important to make lifestyle changes before a pregnancy (Table 4). Adjustment for BMI or smoking did not change the results.

More women in the IG planned to make lifestyle changes after the intervention, especially those with lower education (Table 5). When the question was adjusted for BMI or smoking, no noticeable changes were found in the RRs and corresponding CIs.

**Table 5.** Imagine that you want to get pregnant. Would you take any action before trying to conceive to increase the chances of having a healthy pregnancy and healthy child? Women who answered ‘yes’ before and after the intervention.

	Before		After			
	Intervention	Control	Intervention	Control		
	*n* (%)	*n* (%)	*n* (%)	*n* (%)	Adjusted RR	95% CI
All women	335 (57.4)	346 (58.6)	450 (77.6)	407 (67.9)	1.140	1.063–1.223
Overweight/obesity	116 (58.3)	101 (59.8)	155 (78.7)	111 (64.5)	1.227	1.073–1.403
Smokers	38 (70.4)	34 (70.8)	44 (84.6)	34 (72.3)	1.170	0.947–1.445
Using snuff	27 (90.0)	29 (82.9)	28 (96.6)	25 (71.4)	1.352	1.084–1.685
Binge drinking^a^	99 (62.7)	123 (68.0)	136 (86.6)	136 (75.6)	1.147	1.037–1.269
College/university	98 (55.4)	122 (60.4)	141 (80.1)	136 (67.3)	1.187	1.051–1.340
Elementary school (9 years)	11 (68.8)	9 (52.9)	14 (93.3)	10 (58.8)	1.587	1.042–2.415
Experience of abortion	44 (67.7)	49 (71.0)	51 (81.0)	46 (65.7)	1.214	0.989–1.491
Had given birth	100 (48.8)	103 (52.0)	141 (69.5)	126 (61.8)	1.121	0.973–1.291

RR was adjusted for the same question before the intervention.

### Contraception

There were no differences between the IG and the CG on the use of contraception after the intervention (Table 6). In total, 79% (*n* = 920) were very or fairly satisfied with the current contraceptive method, and those who used the combined hormonal contraceptive pill were the most satisfied (87%, *n* = 217, *P* > 0.001).

**Table 6.** Use of contraceptives at the women’s latest intercourse before and after the intervention.

	Before		After		
	IG	CG	IG	CG	
	*n* (%)	*n* (%)	*n* (%)	*n* (%)	*P*
Combined hormonal contraceptive pill	240 (40.7)	234 (38.9)	241 (40.7)	239 (39.4)	0.654
Condom	113 (19.2)	100 (16.6)	68 (11.5)	59 (9.7)	0.325
Long-acting reversible contraception	128 (21.6)	122 (20.1)	167 (28.2)	159 (26.2)	0.443
Hormonal intrauterine device	39 (6.6)	35 (5.8)	88 (14.9)	78 (12.9)	0.318
Copper intrauterine device	40 (6.8)	40 (6.7)	37 (6.3)	39 (6.4)	0.895
Progestin implant	49 (8.3)	47 (7.8)	42 (7.1)	42 (6.9)	0.912
Combined hormonal contraceptive ring	34 (5.8)	41 (6.8)	41 (6.9)	53 (8.7)	0.241
Progestin-only pill	26 (4.4)	39 (6.5)	33 (5.6)	40 (6.6)	0.458
Progestin-only injection	7 (1.2)	3 (0.5)	13 (2.2)	6 (1.0)	0.095
Other	5 (0.8)	11 (1.8)	11 (1.9)	17 (2.8)	0.278
No method	67 (11.4)	69 (11.4)	45 (7.6)	56 (9.2)	0.307

*P* values are analyzed after the intervention.

In total, 101 women did not use any contraception when they answered Q2, and 57% (*n* = 41 out of 72 who answered the question) were very or fairly satisfied with no contraceptives, 32% (*n* = 23) were neither satisfied nor dissatisfied, and 11.1% (*n* = 8) were fairly or very dissatisfied.

Among these 101 women, 69 women had talked about fertility with a relative, 38 had talked about possibilities of becoming pregnant when it was time, 15 planned their pregnancies or were already pregnant, and 15 were worried about not getting pregnant. Seven had discussed age and fertility, five preconception health, five how to avoid pregnancy, and for two participants it was unclear what they had discussed.

### Age desired for the first and last child

There was no difference between the IG and the CG regarding the age at which the women wanted their first (28 years) and last (33.5 years) child, nor regarding how many children they wanted; on average, the IG and the CG wanted 2.4.

### Pregnancy plan/unplanned pregnancy

Of those who answered Q2, 82% (980/1,195) stated that they had great influence on if and when they will become pregnant. Of those who wished to have children/more children, 65% (516/794) thought it was important to become pregnant according to their own time plans. In response to questions regarding the hypothetical case, ‘If they were to become pregnant today’, 46.5% (557/1,198) responded that they would continue the pregnancy, 26% (311/1,198) would have an induced abortion, and 27.5% (329/1,198) were unsure. Among those who did not want children/more children (229/1,198), 32.3% (*n* = 74) would continue an unplanned pregnancy today, 34.5% (*n* = 79) would have an induced abortion, and 33.2% (*n* = 76) were unsure. Women who were considering induced abortion if they became pregnant did not have a different pattern of contraceptive use compared with others.

### Experience of the intervention

The IG answered three additional questions to evaluate the women’s experiences of the intervention. Three out of four (437/585) had read the brochure. Also, 59.2% (342/577) considered the RLPC as being very or fairly positive, 37.6% (217/577) thought it was neither positive nor negative, 3.1% (18/577) thought it was fairly negative, and no one thought it was very negative. Three out of four women (76%, 443/58) stated that the RLPC should be part of the routine during visits to midwives or other healthcare providers, while 18.2% (106/583) were unsure, and 5.7% (33/583) were negative.

### Spillover effect

To evaluate a possible spillover effect of answering the questionnaires, the women in the CG were asked questions in the follow-up questionnaire about whether they had discussed topics related to our questions with the midwife during the counseling. Half of the women, 50% (307/607), had discussed questions regarding having children in the future, 16% (98/607) had discussed age and fertility. A further 17% (102/607) had discussed the probability of becoming pregnant as a result of unprotected intercourse. There were also 10% who had discussed lifestyle factors that can affect fertility and the chance of a healthy pregnancy. Another 10% had discussed the lifespan of the ovum and the sperm, and 3% had discussed IVF.

## Discussion

We have shown that women’s knowledge about fertility and preconception health was low at baseline; however, the intervention increased their knowledge about fertility and the awareness of factors that affect preconception health, i.e. to stop using tobacco, to refrain from drinking alcohol, to be of normal weight, and to supplement with folic acid before a pregnancy. We found no differences between the IG and the CG with regard to their future reproductive planning and no differences in their choice of contraceptives. The majority of women appreciated the intervention.

We did not expect the IG to achieve 100% knowledge because some women were young and did not plan to have children for many years, while others did not want more children, which may have made them less interested in issues on fertility and preconception health. We also found a ‘spillover effect’ in the CG, suggesting that the study questions about fertility and preconception health may have stimulated women to reflect on these topics during and after the counseling. Based on the questions posed to the women in the CG, we know that some of them also discussed these topics with their midwives.

All midwives working in the outpatient clinics in the study region participated in the data collection, which is a strength because they could recruit participants from different social groups, different education levels, and different occupations. We believe that our results can be generalized to other regions in Sweden, with a similar socioeconomic profile and mean childbirth age.

The response rate for Q2 was 62%, which is satisfactory, as it has gradually become more difficult to recruit people for studies. In public health surveys, the response rate has decreased from 60.8% in 2004 to 47% in 2016, and the response rate was particularly low among young adults (20). In a nationwide study about the use of contraception in 2015, the response rate was 25.3% (21). When comparing attendance rates, we are satisfied with our participation rate.

One limitation is that only Swedish-speaking women were included. The intervention was partly based on direct communication between the midwife and the client, and we did not have financial resources to include interpreters or to print the booklet in other languages. In a previous survey at antenatal clinics, great efforts were made to also include immigrant women, but this was very difficult in spite of having translated questionnaires and interpreters (19). Another limitation was that we included women aged 20–40 years, since we believe that reproductive life planning is of more interest in this specific age range. The non-responders to the follow-up had lower education, a greater proportion were smokers, users of snuff, workers, and born abroad. They had more experience of pregnancies and abortion, but less experience of giving birth. There were more smokers and women with overweight/obesity in the IG; however, we have adjusted for these factors, and the results did not change.

There was notably a low awareness about the impact of lifestyle factors on reproductive health. The women were largely aware that smoking before a pregnancy is unhealthy, but they did not have the same awareness about Swedish snuff, which has similar risks as smoking (22–24). The awareness of the importance of being of normal weight was low among women in our study. In Sweden, 40% are overweight or obese in early pregnancy, and as obesity is increasing in women of fertile ages, prevention of obesity must start years before a pregnancy in order to optimize future pregnancy outcomes (1,25–27).

It was surprising that one-quarter of the women were drinking four glasses or more either once a month or more often, a drinking pattern that women need to recognize as unfavorable and not recommended if planning pregnancy. The knowledge to refrain from drinking alcohol before a pregnancy was also low. The facts that alcohol can cause a miscarriage (28,29) and that fetal alcohol spectrum disorders (FASDs) can occur even in moderate alcohol consumption or binge drinking are important information for women (30–32).

Knowledge about taking folic acid before a pregnancy was lower than expected among the women in our study. Only 30% were aware of the importance of folic acid supplementation when planning a pregnancy, and our results are similar to findings in the study on female university students (11). However, parous women were more aware of this factor than nulliparous, albeit a lower awareness than expected. Parous women have most likely been informed in their earlier pregnancy about the importance of folic acid, but perhaps they did not fully understand that they should start before the pregnancy. Our results show that the Swedish national recommendations about taking folic acid have not reached women of fertile age, and this finding is similar to results in other studies (33,34). Many countries, for example Canada and the USA, have fortified flour with folic acid. The Swedish Agency for Health Technology Assessment and Assessment of Social Services (SBU) carried out a systematic review in 2007 (6) of the scientific evidence on the benefits and risks of fortifying the flour, and they concluded that there was not enough evidence to fortify flour. Their recommendation was that women should be informed about the importance of using folic acid before and during early pregnancy—a strategy that clearly has not been successful, based on the results in this study. Healthcare providers have an important role in informing about folic acid supplementation for women of fertile age. To our knowledge, there have not been any public campaigns in Sweden to increase knowledge about folic acid, as they have had in the Netherlands, but despite their national campaigns the knowledge still remains low (35,36). What kind of strategy or intervention is reasonable to implement in healthcare for improving knowledge is unclear; clearly, improved efforts need to be made both in schools and healthcare in the future.

The first step in making lifestyle changes is to understand what changes are needed and why. Our results show a clear need for lifestyle changes prior to pregnancy in a large proportion of women. The challenge is how these changes in lifestyle could be implemented.

A control question was used in the questionnaire: ‘Is it important to supplement with vitamin C before pregnancy?’ even though there is no recommendation for taking vitamin C. However, a large proportion of women in the IG stated at follow-up that it was important to add vitamin C; perhaps, they mixed this up with folic acid. It could also be that they wanted to answer correctly and, therefore, it might be a weakness of the study.

There was no difference in the choices of contraceptives between the IG and the CG, similar with previous studies (10,11,37). Interestingly, 7.6%–9.2% (*n* = 101) in our study did not use any contraceptive at their latest intercourse, despite having received contraceptive counseling two months earlier. Among these women, 11.1% answered that they were dissatisfied with their contraceptives and could therefore have stopped. Another reason was that some of them had changed their minds and wanted to become pregnant or were already pregnant. Low compliance can also be related to no sexual activity or fear of hormones, side effects, or not finding any suitable method (21,38). Surprisingly many women would continue an unplanned pregnancy, compared to the reported unintended pregnancies worldwide (39).

Because eight out of ten women thought that they can influence when they will become pregnant, and two-thirds answered that it was important to become pregnant at the time they had planned, we assume that many women want to plan their pregnancies.

It has been suggested that healthcare providers should find ways of identifying women who are planning a pregnancy and make use of existing platforms for delivery of preconception care (3). We suggest that contraceptive counseling is an opportunity to talk about preconception health, since three out of four women stated that the RLPC should be part of the routine during visits to midwives. In our study, the midwives gave the women in the IG a specially designed brochure with information about fertility and preconception health, and three out of four women had read this brochure. This evidence-based information has been translated into English and some other languages and is now available on a mobile-friendly website (www.reproduktivlivsplan.se) (40).

The follow-up in our study was conducted after two months, and we have no information on whether the increased knowledge in the IG will lead to a behavioral change when women are planning a pregnancy in the future. The challenge in healthcare is to find prevention strategies that will have long-lasting effects (41). To study the long-term effects of the RLPC intervention, we plan to conduct a follow-up study and evaluate future pregnancy outcomes by linking the study cohort with existing national pregnancy and health registers in Sweden, when the number of future pregnancies in the cohort is sufficiently large.

## Conclusion

The knowledge of fertility and awareness of reproductive health were low at baseline but increased among women in the IG. Most women appreciated the RLPC that can be recommended in contraceptive counseling. However, long-term evaluation of the effects of the intervention on future pregnancies is needed.

## Supplementary Material

Supplemental Material

## References

[1] Stephenson J, Heslehurst N, Hall J, Schoenaker D, Hutchinson J, Cade JE, et al. Before the beginning: nutrition and lifestyle in the preconception period and its importance for future health. Lancet. 2018;391:1830–41. 29673873 10.1016/S0140-6736(18)30311-8 PMC6075697

[2] Fleming TP, Watkins AJ, Velazquez MA, Mathers JC, Prentice AM, Stephenson J, et al. Origins of lifetime health around the time of conception: causes and consequences. Lancet. 2018;391:1842–52. 29673874 10.1016/S0140-6736(18)30312-X PMC5975952

[3] Barker M, Dombrowski SU, Colbourn T, Fall CHD, Kriznik NM, Lawrence WT, et al. Intervention strategies to improve nutrition and health behaviours before conception. Lancet. 2018;391:1853–64. 29673875 10.1016/S0140-6736(18)30313-1 PMC6075694

[4] Socialstyrelsen Statistics on pregnancies, deliveries and newborn infants 2016: Socialstyrelsen, The National Board of Health and Welfare; 2018 [cited 2019 February 7]. Available from: http://www.socialstyrelsen.se/publikationer2018/2018-1-7.

[5] Shawe J, Delbaere I, Ekstrand M, Hegaard HK, Larsson M, Mastroiacovo P, et al. Preconception care policy, guidelines, recommendations and services across six European countries: Belgium (Flanders), Denmark, Italy, the Netherlands, Sweden and the United Kingdom. Eur J Contracept Reprod Health Care. 2015;20:77–87. 25548961 10.3109/13625187.2014.990088

[6] SBU Benefits and risk of fortifying flour with folic acid to reduce the risk of neural tube defects. SBU report no 183. Stockholm: Swedish Council on Health Technology Asseement (SBU); 2007 [in Swedish]. 28876756

[7] Stern J, Salih Joelsson L, Tyden T, Berglund A, Ekstrand M, Hegaard H, et al. Is pregnancy planning associated with background characteristics and pregnancy-planning behavior? Acta Obstet Gynecol Scand. 2016;95:182–9. 26566076 10.1111/aogs.12816 PMC4737297

[8] Moos MK, Dunlop AL, Jack BW, Nelson L, Coonrod DV, Long R, et al. Healthier women, healthier reproductive outcomes: recommendations for the routine care of all women of reproductive age. Am J Obstet Gynecol. 2008;199:280–9. 10.1016/j.ajog.2008.08.060 19081422

[9] Jack BW, Atrash H, Coonrod DV, Moos MK, O’Donnell J, Johnson K The clinical content of preconception care: an overview and preparation of this supplement. Am J Obstet Gynecol. 2008;199:266–79. 19081421 10.1016/j.ajog.2008.07.067

[10] Bommaraju A, Malat J, Mooney JL Reproductive life plan counseling and effective contraceptive use among urban women utilizing title x services. Womens Health Issues. 2015;25:209–15. 25965154 10.1016/j.whi.2015.02.005

[11] Stern J, Larsson M, Kristiansson P, Tyden T Introducing reproductive life plan-based information in contraceptive counselling: an RCT. Hum Reprod. 2013;28:2450–61. 23842564 10.1093/humrep/det279 PMC3748861

[12] Bodin M, Tyden T, Kall L, Larsson M Can reproductive life plan-based counselling increase men’s fertility awareness? Ups J Med Sci. 2018;123:255–63. 30541376 10.1080/03009734.2018.1541948 PMC6327788

[13] Stern J, Bodin M, Grandahl M, Segeblad B, Axen L, Larsson M, et al. Midwives’ adoption of the reproductive life plan in contraceptive counselling: a mixed methods study. Hum Reprod. 2015;30:1146–55. 25771220 10.1093/humrep/dev048 PMC4400198

[14] Tyden T, Verbiest S, Van Achterberg T, Larsson M, Stern J Using the reproductive life plan in contraceptive counselling. Ups J Med Sci. 2016;121:299–303. 27646817 10.1080/03009734.2016.1210267 PMC5098497

[15] Baldwin MK, Overcarsh P, Patel A, Zimmerman L, Edelman A Pregnancy intention screening tools: a randomized trial to assess perceived helpfulness with communication about reproductive goals. Contracept Reprod Med. 2018;3:21. 30574355 10.1186/s40834-018-0074-9 PMC6296099

[16] Hipp SL, Chung-Do J, McFarlane E Systematic review of interventions for reproductive life planning. J Obstet Gynecol Neonatal Nurs. 2019;48:131–9. 10.1016/j.jogn.2018.12.007 30664840

[17] Skogsdal YRE, Karlsson JA, Cao Y, Fadl HE, Tyden TA Contraceptive use and reproductive intentions among women requesting contraceptive counseling. Acta Obstet Gynecol Scand. 2018;97:1349–57. 30007091 10.1111/aogs.13426 PMC6175138

[18] Antikonception - behandlingsrekommendation Information från läkemedelsverket [Contraception - Treament Recommendation. Information from the Swedish Medical Products Agency]. Swedish Medical Products Agency. 2014;25:14–28.

[19] Backhausen MG, Ekstrand M, Tyden T, Magnussen BK, Shawe J, Stern J, et al. Pregnancy planning and lifestyle prior to conception and during early pregnancy among Danish women. Eur J Contracept Reprod Health Care. 2014;19:57–65. 24229390 10.3109/13625187.2013.851183

[20] Folkhälsomyndigheten Nationella folkhälsoenkäten - Hälsa på lika vilkor? Solna: Folkhälsomyndigheten; 2016 [cited 2019 February 7]. Available from: https://www.folkhalsomyndigheten.se/folkhalsorapportering-statistik/statistikdatabaser-och-visualisering/nationella-folkhalsoenkaten/.

[21] Kopp Kallner H, Thunell L, Brynhildsen J, Lindeberg M, Gemzell Danielsson K Use of contraception and attitudes towards contraceptive use in Swedish women–a nationwide survey. PLoS One. 2015;10:e0125990. 25992901 10.1371/journal.pone.0125990 PMC4439158

[22] Baba S, Wikstrom AK, Stephansson O, Cnattingius S Influence of snuff and smoking habits in early pregnancy on risks for stillbirth and early neonatal mortality. Nicotine Tob Res. 2014;16:78–83. 23943841 10.1093/ntr/ntt117

[23] Baba S, Wikstrom AK, Stephansson O, Cnattingius S Changes in snuff and smoking habits in Swedish pregnant women and risk for small for gestational age births. BJOG. 2013;120:456–62. 23190416 10.1111/1471-0528.12067

[24] Baba S, Wikstrom AK, Stephansson O, Cnattingius S Influence of smoking and snuff cessation on risk of preterm birth. Eur J Epidemiol. 2012;27:297–304. 22430122 10.1007/s10654-012-9676-8

[25] Poston L, Caleyachetty R, Cnattingius S, Corvalan C, Uauy R, Herring S, et al. Preconceptional and maternal obesity: epidemiology and health consequences. Lancet Diabetes Endocrinol. 2016;4:1025–36. 27743975 10.1016/S2213-8587(16)30217-0

[26] Hilden K, Hanson U, Persson M, Fadl H Overweight and obesity: a remaining problem in women treated for severe gestational diabetes. Diabet Med. 2016;33:1045–51. 27172974 10.1111/dme.13156 PMC5089567

[27] Marchi J, Berg M, Dencker A, Olander EK, Begley C Risks associated with obesity in pregnancy, for the mother and baby: a systematic review of reviews. Obes Rev. 2015;16:621–38. 26016557 10.1111/obr.12288

[28] Andersen AM, Andersen PK, Olsen J, Gronbaek M, Strandberg-Larsen K Moderate alcohol intake during pregnancy and risk of fetal death. Int J Epidemiol. 2012;41:405–13. 22253313 10.1093/ije/dyr189

[29] Feodor Nilsson S, Andersen PK, Strandberg-Larsen K, Nybo Andersen AM Risk factors for miscarriage from a prevention perspective: a nationwide follow-up study. BJOG. 2014;121:1375–84. 24548778 10.1111/1471-0528.12694

[30] Knudsen AK, Skogen JC, Ystrom E, Sivertsen B, Tell GS, Torgersen L Maternal pre-pregnancy risk drinking and toddler behavior problems: the Norwegian Mother and Child Cohort Study. Eur Child Adolesc Psychiatry. 2014;23:901–11. 25053124 10.1007/s00787-014-0588-x PMC4186966

[31] Alvik A, Aalen OO, Lindemann R Early fetal binge alcohol exposure predicts high behavioral symptom scores in 5.5-year-old children. Alcohol Clin Exp Res. 2013;37:1954–62. 23888929 10.1111/acer.12182

[32] Williams JF, Smith VC; Committee on Substance Abuse Fetal alcohol spectrum disorders. Pediatrics. 2015;136:e1395–406. 26482673 10.1542/peds.2015-3113

[33] Lampic C, Svanberg AS, Karlstrom P, Tyden T Fertility awareness, intentions concerning childbearing, and attitudes towards parenthood among female and male academics. Hum Reprod. 2006;21:558–64. 16293651 10.1093/humrep/dei367

[34] Bestwick JP, Huttly WJ, Morris JK, Wald NJ Prevention of neural tube defects: a cross-sectional study of the uptake of folic acid supplementation in nearly half a million women. PLoS One. 2014;9:e89354. 24586711 10.1371/journal.pone.0089354 PMC3929694

[35] de Walle HE, de Jong-van den Berg LT Ten years after the Dutch public health campaign on folic acid: the continuing challenge. Eur J Clin Pharmacol. 2008;64:539–43. 18213474 10.1007/s00228-007-0446-6 PMC2668616

[36] Temel S, Erdem O, Voorham TA, Bonsel GJ, Steegers EA, Denktas S Knowledge on preconceptional folic acid supplementation and intention to seek for preconception care among men and women in an urban city: a population-based cross-sectional study. BMC Pregnancy Childbirth. 2015;15:340. 26684337 10.1186/s12884-015-0774-y PMC4684618

[37] Fooladi E, Weller C, Salehi M, Abhari FR, Stern J Using reproductive life plan-based information in a primary health care center increased Iranian women’s knowledge of fertility, but not their future fertility plan: a randomized, controlled trial. Midwifery. 2018;67:77–86. 30267937 10.1016/j.midw.2018.09.011

[38] Fennell J “And isn’t that the point?”: pleasure and contraceptive decisions. Contraception. 2014;89:264–70. 24332430 10.1016/j.contraception.2013.11.012

[39] Sedgh G, Singh S, Hussain R Intended and unintended pregnancies worldwide in 2012 and recent trends. Stud Fam Plann. 2014;45:301–14. 25207494 10.1111/j.1728-4465.2014.00393.x PMC4727534

[40] Ekstrand Ragnar M, Niemeyer Hultstrand J, Tyden T, Larsson M Development of an evidence-based website on preconception health. Ups J Med Sci. 2018;123:116–22. 29909720 10.1080/03009734.2018.1476423 PMC6055744

[41] Sylvest R, Koert E, Vittrup I, Petersen KB, Andersen AN, Pinborg A, et al. Status one year after fertility assessment and counselling in women of reproductive age – a qualitative study. Ups J Med Sci. 2018;123:264–70. 30539672 10.1080/03009734.2018.1546243 PMC6327567

