# Supplementary material for: An intervention in contraceptive counseling increased the knowledge about fertility and awareness of preconception health—a randomized controlled trial
Source: Ups J Med Sci. 2019 Sep 9;124(3):203–12. doi: 10.1080/03009734.2019.1653407 (PMC6758707; doi:10.1080/03009734.2019.1653407)
Supplement: Supplemental Material [file IUPS_A_1653407_SM5104.docx]

Supplement I

The aim with contraceptive counselling is to prevent unwanted pregnancies and save the fertility until a pregnancy is wanted. It shall also promote sexual and reproductive health.

The counselling should include a careful history of:

- Earlier and ongoing diseases and its treatments.
- Medication including herbal.
- Allergies.
- Use of tobacco, alcohol and drugs.
- Occurrence in first-degree relatives (parent or siblings) of venous thromboembolism, cardiovascular disease or other serious disease.
- Previous pregnancies.
- Previous experience of contraception.
- Menstruation.
- Need to hide the use of contraceptives.

Investigation/sampling

- Weight, length, BMI and blood pressure.
- A gynecological examination can be offered, but not necessary.
- Offer wet smear to women who have not attended the screening program.
- Offer test for chlamydia and other sexual transmitted infections.

Counselling about contraception, how to use them and side effects.
